# Deep learning for blind structured illumination microscopy

**DOI:** 10.1038/s41598-022-12571-0

**Published:** 2022-05-21

**Authors:** Emmanouil Xypakis, Giorgio Gosti, Taira Giordani, Raffaele Santagati, Giancarlo Ruocco, Marco Leonetti

**Affiliations:** 1 Center for Life Nano- and Neuro-Science, Istituto Italiano di Tecnologia, Viale Regina Elena 291, 00161 Rome, Italy; 2 D-TAILS srl, 00161 Rome, Italy; 3 Dipartimento di Fisica, Sapienza Università di Roma, Piazzale Aldo Moro 5, 00185 Rome, Italy; 4 Quantum Engineering Technology Labs, University of Bristol, Bristol, BS8 1FD UK; 5 Quantum Lab, Boehringer-Ingelheim, Doktor-Boehringer-Gasse 5-11, 1120 Wien, Austria; 6 Soft and Living Matter Laboratory, Institute of Nanotechnology, Consiglio Nazionale delle Ricerche, 00185 Rome, Italy

**Keywords:** Imaging and sensing, Applied optics, Optical physics, Optical techniques

## Abstract

Blind-structured illumination microscopy (blind-SIM) enhances the optical resolution without the requirement of nonlinear effects or pre-defined illumination patterns. It is thus advantageous in experimental conditions where toxicity or biological fluctuations are an issue. In this work, we introduce a custom convolutional neural network architecture for blind-SIM: BS-CNN. We show that BS-CNN outperforms other blind-SIM deconvolution algorithms providing a resolution improvement of 2.17 together with a very high *Fidelity* (artifacts reduction). Furthermore, BS-CNN proves to be robust in cross-database variability: it is trained on synthetically augmented open-source data and evaluated on experiments. This approach paves the way to the employment of CNN-based deconvolution in all scenarios in which a statistical model for the illumination is available while the specific realizations are unknown or noisy.

## Introduction

In optics, the diffraction limit is the minimum distance at which two objects can be identified and it depends on wavelength and numerical aperture^1^. It is possible to surpass the diffraction limit and improve the image contrast by removing or reversing the effects of diffraction and other sources of noise^2^ with deconvolution methods. The performance of these methods depends on both the knowledge of the experimental conditions and the imaged sample properties. For example, sparsity of molecular emitters induced by photoactivation or photobleaching enables the observer to achieve a more-than-ten times resolution enhancement^3–5^ thanks to single molecule localization techniques. Another popular technique, Structured Illumination Microscopy (SIM)^6^, improves the resolution up to a factor 2 by exploiting multiple spatially periodic illumination patterns. However, this resolution enhancement rapidly degrades if patterns are instable or if knowledge of the spatial illumination profile is imprecise. Indeed, image reconstruction and deconvolution may be performed also with unknown illumination patterns^7–11^. These blind-SIM techniques are not subject to pattern evaluation errors, such as misalignment and repeatability issues, and are immune to aberrations on the illumination path^12^.

Blind-SIM deconvolution methods, such as Scattering Assisted Imaging (SAI)^11^, often requires complex and computationally expensive deconvolution algorithms. Luckily, the advent of Neural Networks (NN) provided new solutions for image analysis. NN are capable of reducing the computational time for deconvolution and improve image resolution^13^. Typically they exploit dictionary learning and the upsampling of sparse representations^14,15^ that efficiently remove complex blurring^16,17^. In Microscopy, Deep Neural Networks can tackle the inverse imaging problem^18^, deconvolve images from different fluorescence microscopy modalities such as wide-field fluorescence or confocal microscopy^19^, and efficiently localize single molecules^20,21^ or dimers^22^. In SIM, a NN enables a substantial reduction of the required frames number by using a multi-channel architecture^23^, and can provide an improved noise resilience^24^. To the best of our knowledge neural networks have never been applied to blind-SIM.

**Figure 1:**
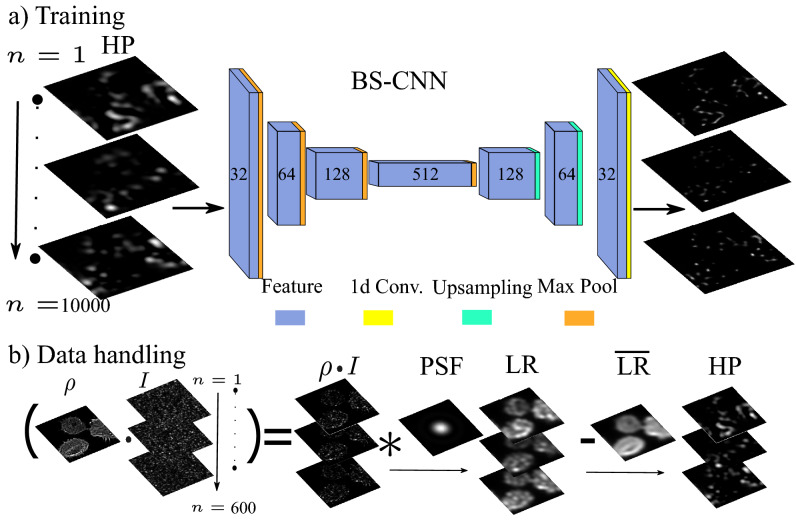
BS-CNN scheme: (**a**) We train the CNN with HP frames so that each illumination and each fluorescent density appears only once and minimizes the loss by comparing with the corresponding $\rho \cdot I$ image. The BS-CNN architecture: for the encoder-decoder architecture we use two-dimensional convolutional layers of size 3 $\times$ 3 followed by an element-wise ReLU non-linearity. The feature number is first increased (encoder) by a factor of two from 32 to 512 while the image size is decreased by a 4 $\times$ 4 max pool layer. Then the opposite procedure is applied (decoder) up to the original image size. Finally, a one-dimensional convolutional layer is applied to produce a single-channel image. (**b**) Data handling: a ground truth $\rho$ neuron image is selected from an open microscopy repository^25^; the ground truth image is illuminated 600 times by different speckle realizations *I*, producing 600 high-frequency images; we convolve with an Airy disk Point spread function (PSF) of resolution $\eta$ times bigger than the speckle correlation size and obtain 600 low-resolution frames corresponding to the same GT. By subtracting the low-resolution mean $\overline{\text {LR}}$ from each low-resolution frame LR and keeping only the high positive part we obtain the HP images.

In this work, we design, test, and validate a Convolutional Neural Network for the reconstruction of blind SIM deconvolutions: the BS-CNN. This approach enables to deconvolve images in a few seconds and to surpass other state of the art blind-SIM deconvolution algorithms in performance such as^7,11^.

## Results

### CNN training

BS-CNN deconvolves images by employing an encoder-decoder architecture that has been adapted and modified for bind SIM. Encoder–decoder network architectures are widely used in biology applications such as image deconvolution^21^ or cell counting^26^. They are very easy to adapt and train if compared with the more sophistcated and potentially more powerful UNET architecture^27,28^ They are based on an information theory framework, in which a first encoding step generates a high-dimensional dense feature representation of the input, and a second decoding step upsamples the information in the desired representation^29^. In the encoding stage (see Fig.1 panel a), BS-CNN encodes the input in a dense feature representation by applying two-dimensional $3 \times 3$ convolutional layers of feature size 32, 64, 128 and 512 respectively, then followed by an element wise ReLU layer and a Max Pool layer. In the decoding stage, the 512 dense features are decoded to the original input size by three $3 \times 3$ two-dimensional deconvolutional layers of feature size 128, 64 and 32 respectively, followed by an element wise ReLU layer and an upsampling layer. The aim of this CNN is to reconstruct, from low-resolution fluorescence images, a high-resolution fluorophores-density spatial distribution $\rho$. To perform the training, we designed an original protocol to augment images from a publicly available open-source data-set^25^ with synthetically generated illumination patterns. More precisely, we started from fluorescence microscopy images of biological targets, which we used as high resolution images target ground truth (GT). They represent the spatial distribution of the fluorophore molecules $\rho$. We pre-processed images from the repository by randomly extracting 10000 patches, each with a field of view 11 times larger than the Point Spread Function (PSF) of the optical system. We used 7500 of these patches for training, 2000 for validation, and 500 for testing. Then we “illuminated” the patches with speckle patterns as required for the standard blind-SIM approach. Speckle patterns^30^, which are light structures generated on a laser beam reflected by a rough surface, occur in the presence of disordered phase patterns. We generated numerically synthetic speckle patterns $I(\mathbf {r})$ employing a model based on summing plane waves with random phases, random amplitudes, and a cuttoff spatial frequency that enables us to control the average speckle grain size of $d_{sp}$ (see Supplementary Materials). In a typical blind-SIM experiment, a few hundred of different illuminations replicas ($N=600$) are acquired to extract a high-resolution image. High-resolution fluorescent signal data are obtained simply by multiplying $I\cdot \rho$. Note that, to ensure generality, each patch and illumination appears only once in the dataset. This syntactically augmented data-set contains sufficient variability to incorporate the physical properties of the illumination patterns in the deconvolution process. The low-resolution images are then generated from the high-resolution ones applying a blurring convolutional kernel, with the size of the collection point spread function $d_{PSF}$. This convolution operation mimics the information loss effect due to a collection objective with a limited numerical aperture (see Supplementary Materials). The ratio between $d_{PSF}$ and $d_{sp}$

1 $$\begin{aligned} \eta = \frac{d_{PSF}}{d_{sp}} \end{aligned}$$

is the parameter driving the effectiveness of the deconvolution. In a standard experiment, performed with an objective for both illuminated and collected light, focusing and collection PSF are identical (apart a small factor due to wavelength differences), that is $d_{PSF}=d_{sp}$. However, if two different optics are employed, $d_{PSF}$ and $d_{sp}$ could be different (see Supplementary Materials). In fact the Scattering Assisted Imaging technique^11^ takes advantage of a reduced $d_{sp}$ to improve resolution. Thus we studied the general case in which $d_{sp}<d_{PSF}$ that is $\eta \ge 1$.

Before submitting data to the CNN for training the High Positive part of the Intensity of the signal HP is extracted from the low-resolution fluorescence signal

2 $$\begin{aligned} HP_n = (LR_n- LR)^{+}, \end{aligned}$$

where $()^{+}$ denotes that we keep only the matrix elements that are above zero and $LR_n$ is a set of *N* Low Resolution images with an average *LR*. This data handling sparsifies the low-resolution images so that the algorithm makes use of the sparse nature of high intensity speckles (see^11^ and Supplementary Materials). The model is trained using the adam^31^ algorithm, with exponential decay rate for the 1st moment $\beta _1=0.9$, and exponential decay rate for the 2nd moment $\beta _2=0.999$. We complete training after 120 epochs with a learning rate $lr= 0.001$ and we use as a loss the structural similarity index SSIM (see Supplementary Materials and^32^). we stop the training when the validation loss is not improving. We found that the best result is obtained when we use a batchsize of 32.

**Figure 2:**
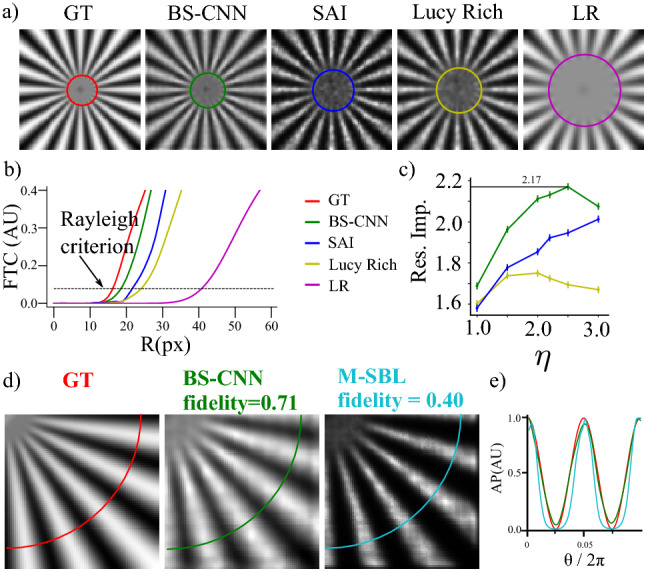
Resolution measurement: (**a**) A Siemens star of density $\rho = 1+ cos20 \theta$ (field of view is 13 times $d_{PSF}$). From left to right: the ground truth GT, the LR deconvolved with BS-CNN, with SAI, and with Lucy Richardson and the low resolution LR. All the deconvolution have been obtained with $N=1000$; The last frame is the Low-Resolution one (LR) obtained from the GT convolving it with the PSF of FHWM $d_{PSF}$ ($\eta = 2.5$). The radii of circles highlighted in the figure correspond radius at which the Siemens star rays are resolved. (**b**) The Fourier transform contrast FTC versus the radius *R* . The dashed line represents the Rayleigh criterion. (c) Resolution improvement of the BS-CNN, Lucy Richardson and SAI as a function of $\eta$ with error-bars. The maximum resolution improvement is 2.17. **(d)** From left to right: quarter of the Siemens star for the GT, BS-CNN, and M-SBL (**e**) azimuthal profiles from (**d**).

### Resolution measurement and fidelity

**Figure 3:**
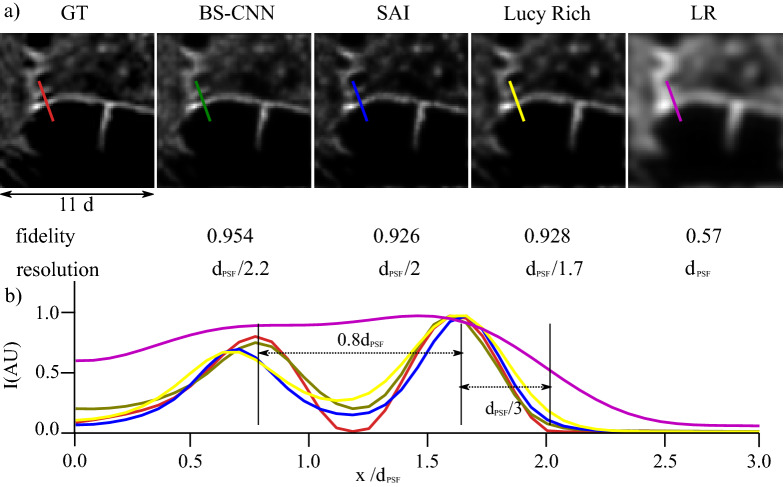
Comparison of different algorithms. In panel (**a**) the images from left to right are the ground truth GT, BS-CNN, SAI and Lucy Richardson (Lucy Rich.) deconvolutions of the low-resolution LR. All deconvolutions are for *N* =1000 and $\eta$=2.5. Below the images we label the *Fidelity* and the resolution expressed in terms of the PSF FWHM $d_{PSF}$. In panel (b) we show the line profile for each algorithm which has length three times $d_{PSF}$. The field of view for each image is 11 $d_{PSF}$.

In Figs. 2 and 3 we show the performance of the BS-CNN algorithm averaged over 1000 illuminations and compare it with the SAI algorithm, a custom local search deconvolution algorithm that exploits the properties of the illumination statistics (see Supplementary Materials and^11^ ) and and with the Lucy Richardson algorithm, fixing $\eta =2.5$ .

In Fig.2a we show the Siemens Star object: a standard to extract image resolution. It has been degraded to obtain the Low-Resolution image (image on the right, LR) and then super resolved with (from left to right ) BS-CNN, SAI, and Lucy Richardson. The colored rings centered on the each star represent the radial value R where the Rayleigh criterion is satisfied. BS-CNN show the the best resolution. Resolution enhancement is indeed further quantified in Fig. 2b, where we show the Fourier transform contrast FTC (see Supplementary Materials and^12^) of the azimuthal profiles for different radii R, where the Rayleigh criterion is satisfied when FTC= 0.08. The BS-CNN (Green Curve) provides the best performance over the investigated algorithms, since it achieves a higher FTC at all radii. In particular for $\eta$=2.5 it achieves a Resolution Improvement (see Supplementary Materials) of 2.17, higher than the other algorithms. This is further confirmed by panel2c where we report the resolution improvement versus the $\eta$ parameter. The BS-CNN (green Curve) always outperforms other algorithms. Additionally BS-CNN produces images which are less affected by residual “granularity”, i.e. from speckle sized intensity fluctuations which are due to the limited number of illuminations. In panel 2d we test *Fidelity* (i.e. the capability to provide artifact free results) of BS-CNN. The *Fidelity* is obtained by computing the structural similarity index (^32^ , see Supplementary Materials) between deconvolved images and the ground truth. *Fidelity* is equal to 1 if the image resulting from deconvolution is identical to the *GT* while it is close to 0 for a poorly performing algorithm. BS-CNN provides a *Fidelity* of 0.71. This value has to be compared to other blind-SIM deconvolution techniques such as M-SBL^7,33^ which, even providing slightly sharper images, deform the shape of the Siemens Star. The higher fidelity to the GT of BS-CNN is also confirmed by intensity profiles in Fig. 2 panel e providing a comparison of the profiles obtained with the different algorithms to the GT.

### Validation in experimental data

In Fig. 3 we report results obtained applying BS-CNN to the analysis of live cells image, together with the degree of *Fidelity* of each technique. BS-SIM achieves a *Fidelity* of 95.4 per cent, in contrast to SAI and Lucy Richardson algorithm which achieve 92.6 and 92.8 per cent respectively. In particular, in the line profiles of the same figure (Fig. 3 panel b), we show two objects lying closer than the PSF resolution $d_{PSF}$ so that they are not resolved in the low-resolution image. BS-CNN finds accurately both the position of the maxima and their relative intensity, while SAI and Lucy Richardson misplace the left maximum and retrieve it with a lower intensity.

**Figure 4:**
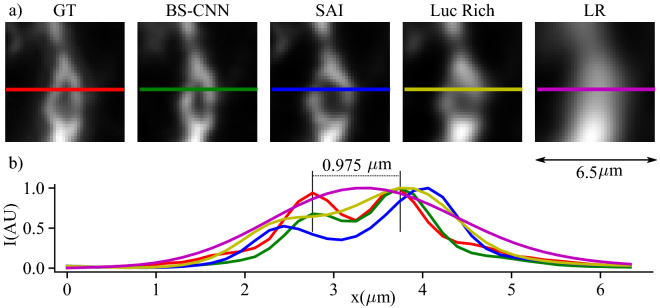
Performance on experimental measurements: the output of the BS-CNN averaged over 600 low-resolution frames for mouse neurons prepared with Alexa Fluor 533 fluorophores along with the line profiles and comparison with the standard SAI and Lucy Richardson deconvolution. The field of view is 6.50 $\upmu$m. The low resolution image has a resolution of $2.37 \upmu$m. BS-CNN achieves an image resolution of $0.975 \upmu$m.

Finally, we validate the BS-CNN approach on fully experimental data in Fig. 4, where speckles are generated employing a Digital Micromirror Device (DMD), with a random pattern controlling the illumination laser wavefront. Different illuminations are successively delivered to the sample by recasting the random pattern. To control the $\eta$ parameter, the collection PSF (and in particular the $d_{PSF}$ parameter) has been tuned with an optical iris placed between the collection objective and the eyepiece^11^. In Fig. 4 we again compare BS-CNN, SAI and Lucy Richardson for $\eta =2.5$ (maximum distance between distinguishable objects of $2.37 \,\upmu$m) and with $N=$ 600 illuminations. As we observe from the plot, BS-CNN can resolve objects up to a distance of 0.975 $\mu$m which is 2.5 times smaller than the low-resolution object outperforming Lucy Richardson and SAI.

## Discussion

In summary we trained and studied a CNN able to deconvolve blind-SIM image stacks in a wide range of $\eta$ values thus capable to be effective in different experimental configurations. BS-CNN has been trained employing images of biological samples (cell images) taken from a repository and “illuminated” by synthetic optical patterns generated with a model for speckles, but also tested on real experimental data on biological samples. Even though the testing conditions are very different than those of the training, the BS-CNN confirms its effectiveness and robustness to data variability. We found that the BS-CNN outperforms other blind-SIM deconvolution algorithms and achieves an average resolution improvement with respect to the low-resolution images of 2.17, while it can reach up to a resolution improvement of 2.5 in specific spots. This improved performance is probably due to the neural networks generalization capability. The BS-CNN also produces images closer to the ground truth, as demonstrated quantitatively by the *Fidelity* parameter, indeed avoiding granularity or nonlinear effects. Moreover, we show an original approach for the CNN training, coupling a model of the illumination with experimental data. This strategy may be exported beyond microscopy, in all experimental configurations in which a modeling of the illumination is available, thus extending the data set available for training without degrading the final performance or the *Fidelity*.

## Methods

### Microscope details

A continuous-wave (CW) laser emits light at the wavelength $$\lambda = 638\,nm$$. A first telescope enlarges the beam’s size with two lenses respectively of focal lengths $$f_1 = 7\,$$ cm and $$f_2 = 20 \, $$cm. Then the light is modulated by a Digital Micromirror Device (DMD) by a random binary mask to generate different speckle illuminations. This plane is imaged through a 4*f* lens system, made by a lens $$f = 20\, cm$$, and an objective (numerical aperture $$NA = 0.75$$), to the sample. The objective collects the reflected light and the fluorescence that are then imaged by a third lens ($$f = 20 $$cm) to the camera (ORCA-Flash4.0 V3 Digital CMOS camera). A dichroic mirror and a long-pass spectral filter select the fluorescence signal. The camera’s pixel size is $$6.5\, \upmu $$ m, while the overall magnification is 40. The spatial resolution of the microscope can be degraded by means of an iris placed in front of the third lens. The sample is composed by retina neurons prepared with Alexa Fluor 533 fluorophores. The fluorescence measurements have been performed by illuminating the sample with 600 speckles illuminations and an exposure time of $$0.2\,$$ s.

In SAI each low resolution frame $LR_n(\mathbf {r})$ is approximated with a synthetic frame $g_n(\mathbf {r})$ composed by a sum of *K* Airy disks ${\tilde{h}}(\mathbf {r})$ that have a size of $d_{sp}$ and are located in random positions $\mathbf {r}_{nk}$.

### Scattering assisted imaging (SAI) deconvolution

The task of the deconvolution algorithm is to find the fluorophore density $\rho (\mathbf {r})$ given the low resolution set $LR_n(\mathbf {r})$. In SAI each $LR_n(\mathbf {r})$ is approximated with a synthetic frame $g_n(\mathbf {r})$ composed by a sum of *K* Airy disks ${\tilde{h}}(\mathbf {r})$ that have a size of $d_{sp}$ and are located in random positions $\mathbf {r}_{nk}$,

3 $$\begin{aligned} g_n(\mathbf {r}) =\sum \limits _{k=1}^{K} P_{nk} {\tilde{h}}(\mathbf {r};\mathbf {r}_{nk}). \end{aligned}$$

The distribution of $P_{nk}$ is a properly normalized multiplying factor respecting an exponential distribution. SAI finds the most probable configuration for the support $\mathbf {r}_{nk}$ for each frame by a local search process that minimizes the mean absolute error between the $HP_n$ and the convoluted frame of $g_n(\mathbf {r})$

4 $$\begin{aligned} F = \sum \limits _{\mathbf {r}} \left| (g_n(\mathbf {r})*h(\mathbf {r}))- HP_n(\mathbf {r})\right| , \end{aligned}$$

where $*$ is the convolution operator. The final output of the algorithm $G(\mathbf {r})$ is an average over the ensemble

5 $$\begin{aligned} G(\mathbf {r}) = \sum \limits _{n=1}^{N} \frac{g_n(\mathbf {r})}{N}. \end{aligned}$$

## Supplementary Information

Supplementary Information.
