# Supplementary material for: Deep learning for blind structured illumination microscopy
Source: Sci Rep. 2022 May 21;12:8623. doi: 10.1038/s41598-022-12571-0 (PMC9124205; doi:10.1038/s41598-022-12571-0)
Supplement: Supplementary file 1 — Supplementary Information. [file 41598_2022_12571_MOESM1_ESM.pdf]

Supplementary Information: Deep Learning for blind structured illumination microscopy

Emmanouil Xypakis[1,2*], **Giorgio Gosti**[1,6], **Taira Giordani**[1,3], **Raffaele Santagati**[4,5], **Giancarlo Ruocco**[1], **and Marco Leonetti**[1,2,6]

[1]Center for Life Nano- & Neuro-Science, Istituto Italiano di Tecnologia, Viale Regina Elena 291, 00161, Rome, Italy
[2]D-TAILS srl, Rome, 00161, ITALY
[3]Dipartimento di Fisica, Sapienza Università di Roma, Piazzale Aldo Moro 5, I-00185 Roma, Italy
[4]Quantum Engineering Technology Labs, University of Bristol, BS8 1FD, UK
[5]Boehringer-Ingelheim Quantum Lab, Wien, Austria.
[6]Soft and Living Matter Laboratory, Institute of Nanotechnology, Consiglio Nazionale delle Ricerche, I-00185 Rome, ITALY
[*]Corresponding author: Emmanouil.Xypakis@iit.it

ABSTRACT

This document provides supplemental information to "Deep Learning for blind structured illumination microscopy". It is composed of four section: 1. Numerical Speckles and Convolution Kernels generation; 2. Blind SIM Deconvolution models; 3. Comparison with other techniques: Random Illumination Microscopy (RIM); 4. Resolution Measurement and Robustness to Noise

1 Numerical Speckles and Convolution Kernel Generation

In the experiment, the speckle illumination patterns, I are generated by exploiting a laser, reflected by a properly shaped DMD[1] or exploiting a strong scattering of refractive index n_s, generating a speckle with correlation FWHM size $d_{sp} = \frac{0.61\lambda_{exc}}{NA_{Eff}}$ where λ_{exc} is the excitation wavelength and NA_{Eff} is an effective numerical aperture depending on n_s. To numerically produce the illumination I numerically we Fourier transform the exponential of a phasor after applying a low pass filter as illustrated in Supplementary Fig. S. 1 and multiply by the conjugate

$$I(\mathbf{r}) = FFT(e^{i\phi_{rdm}(\mathbf{r})}) \cdot \widehat{FFT(e^{i\phi_{rdm}}(\mathbf{r}))}, \tag{1}$$

where FFT denotes the Fourier transform. and $\widehat{FFT}$ denotes the complex conjucation. To generate the point spread function (PSF) $h(\mathbf{r})$ we use an Airy disk of resolution $d_{PSF} = 0.61\frac{\lambda_{em}}{NA}$ where λ_{em} is the emission wavelength of the fluorophores and NA is the numerical aperture of the microscope. In the Fourier space the η parameter in the main text is defined as

$$k_{ill} = \eta k_{PSF}, \tag{2}$$

where k_{ill} is the illumination cut-off frequency, while the k_{PSF} is the collection cut off frequency as illustrated for the speckles in Supplementary Fig. S. 1.

2 Comparison with other techniques: Random Illumination Microscopy (RIM)

Another popular deconvolution algorithm is the Random Illumination Microscopy (RIM) that forms the variance of the speckled images and by using the spatial characteristics of the illumination improves the resolution[2]. We compare BS-CNN with RIM performance for a set of 800 Low resolution images in Supplementary Fig. S. 3 for $eta = 2$. BS-CNN outperfroms marginaly RIM according the the SSIM and PSNR and resolution improvement indices while RIM reconstruction come along with non-linearity artifacts.

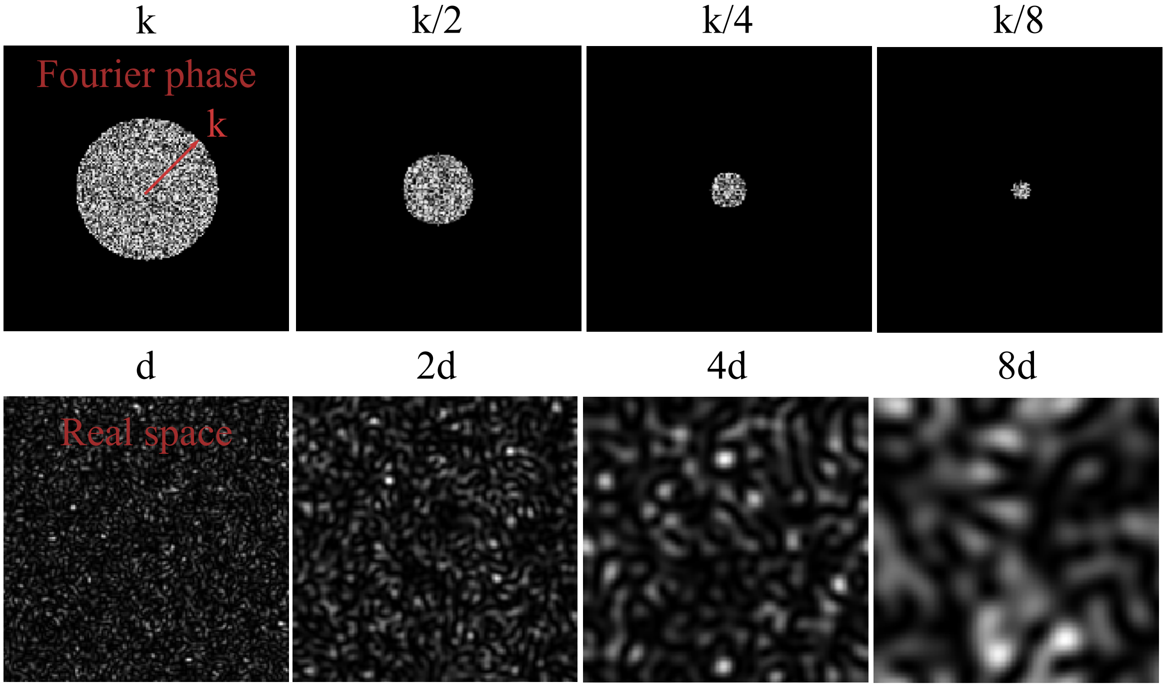

Supplementary Figure S 1. Numerical speckle generation: we show the generation of speckles of different sizes d, 2d, 4d, 8d. The speckles are generated by summing all the plane waves, each carrying a random phase, below a cutoff frequency k, k/2, k/4, k/8 respectively.

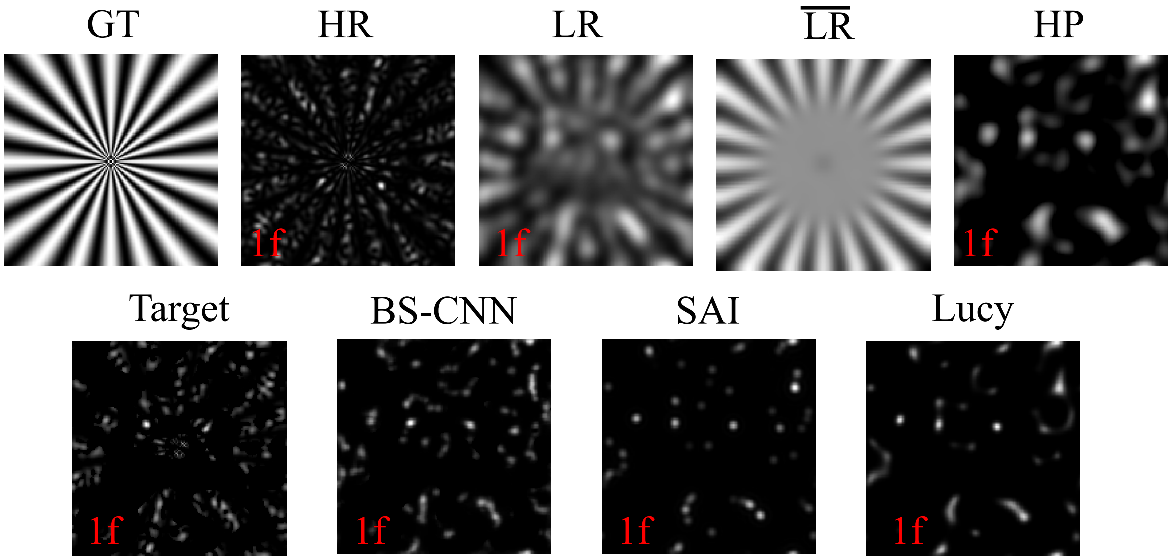

Supplementary Figure S 2. The deconvolution process: GT is the ground truth, HR is the High Resolution image produced by illuminating the GT with one speckle illumination (one frame: 1f), LR is the low-resolution image, $\overline{LR}$ is the low-resolution mean image, HP is the high intensity part of LR, the target image is the ideal configuration that our deconvolution algorithm produces from the HP part of the LR frame, BS-CNN is the single frame output of the BS-CNN, SAI is the single frame output of SAI, Lucy is the single frame output of the Lucy Richardson algorithm applied in the HP part of the data.

a)

SSIM	0.878	0.876
PSNR	18.761	18.665
Res. Imp.	2.049	1.967

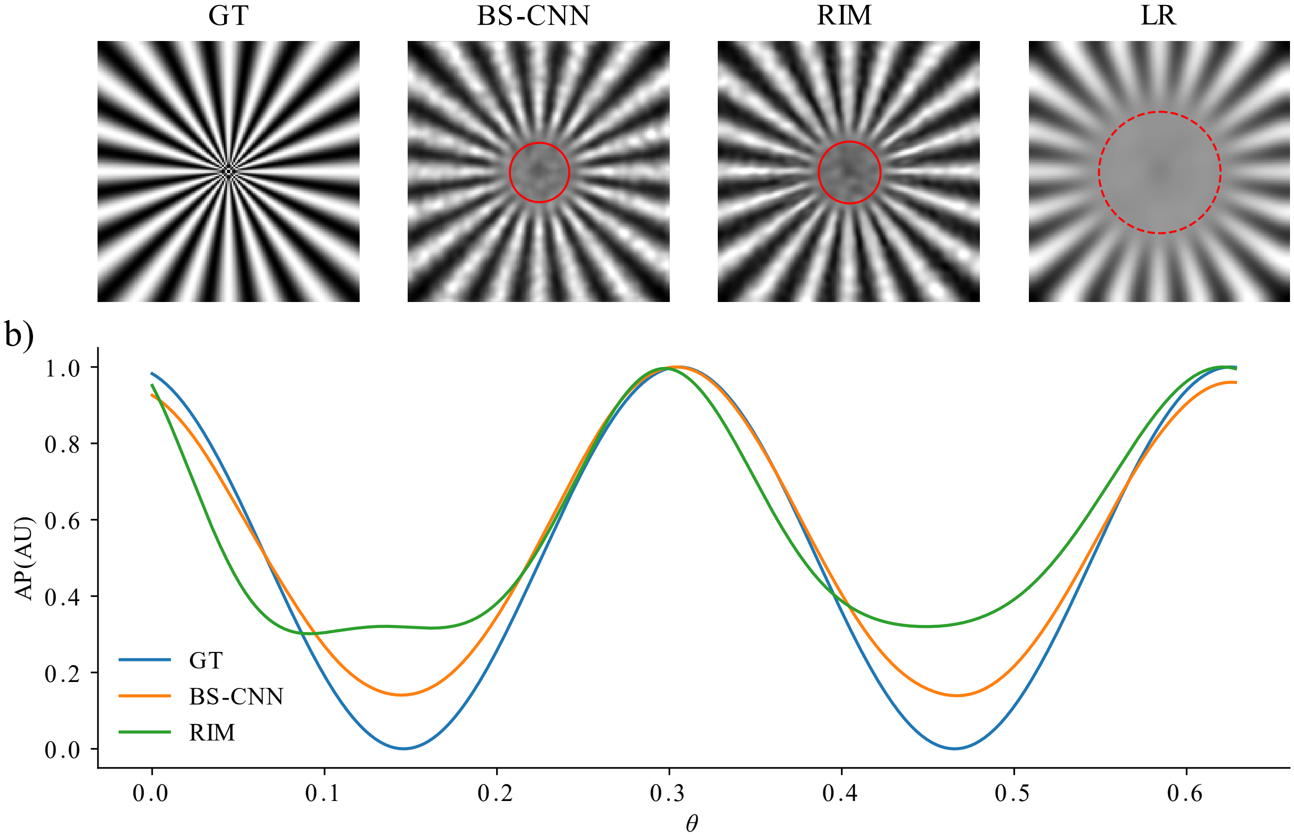

Supplementary Figure S 3. The comparisson of BS-CNN with respect to RIM algorithm on artificial data for $\eta = 2$. a) From left to right: the ground truth GT; the BS-CNN reconstruction; the RIM reconstruction; the low resolution image averaged for 800 low resolution frames. The red circles correspond to the Rayleigh criterion. Above the figures are the metrics structural similarity index SSIM and peak signal to noise ration PSNR. b) The azimuthal profile AP for GT, BS-CNN and RIM for a circle with radious equal to the low resolution criterion (red dashed circle in LR)

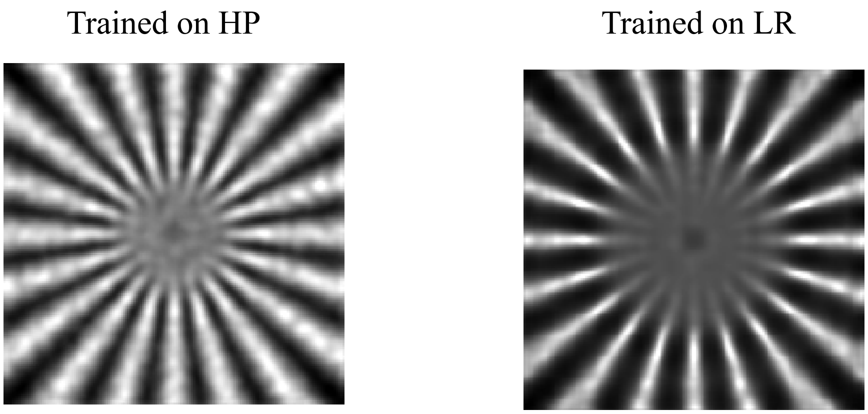

	Trained on HP	Trained on LR
SSIM	0.88	0.62
PSNR	18.76	12.69
Res. Imp.	2.05	1.84

Supplementary Figure S 4. The comparison of BS-CNN when trained with the LR data as an input or trained with HP as an input. The results are averaged over 800 low resolution frames. Below the figures are the metrics structural similarity index SSIM and peak signal to noise ratio PSNR.

3 Details on the training of the CNN

3.1 Loss function and training details

The network is trained by using the structural similarity index (SSIM)[3] as the loss function. We use the SSIM also as a measure of fidelity for the outputs of the deconvolution models

$$Fidelity = SSIM. \tag{3}$$

For two images $U_\tau(\mathbf{r})$ and $S(\mathbf{r})$ SSIM is defined as

$$SSIM(U,S) = \frac{(2\mu_U\mu_S + c_1)(2\sigma_{US} + c_2)}{(\mu_U^2 + \mu_S^2 + c_1)(\sigma_U^2 + \sigma_S^2 + c_2)}, \tag{4}$$

where

$$\begin{aligned}
\mu_U &= \sum_{\mathbf{r}} w(\mathbf{r})U(\mathbf{r}), \\
\mu_S &= \sum_{\mathbf{r}} w(\mathbf{r})S(\mathbf{r}), \\
\sigma_U &= (\sum_{\mathbf{r}} w(\mathbf{r})(U(\mathbf{r}) - \mu_U)^2)^{1/2}, \\
\sigma_S &= (\sum_{\mathbf{r}} w(\mathbf{r})(S(\mathbf{r}) - \mu_S)^2)^{1/2}, \\
\sigma_{US} &= \sum_{\mathbf{r}} w(\mathbf{r})(U(\mathbf{r}) - \mu_U)(S(\mathbf{r}) - \mu_S),
\end{aligned} \tag{5}$$

with $c_1 = 0.01$, $c_2 = 0.03$, and $w(\mathbf{r})$ is an average 11x11 window gaussian filter of $\sigma = 1.5$. In the training we also use the Mean Square Error between two images, which is defined as

$$MSE(U,S) = \sum_{\mathbf{r}} \frac{(U(\mathbf{r}) - S(\mathbf{r}))^2}{L^2}, \tag{6}$$

where L is the field of view of the square image.

3.2 Training strategy

After training on both the low resolution images and on the HP Supplementary Fig. S 2 we observed 4 that the network performs better when trained on the HP. The encoder-decoder architecture handles better sparse data.

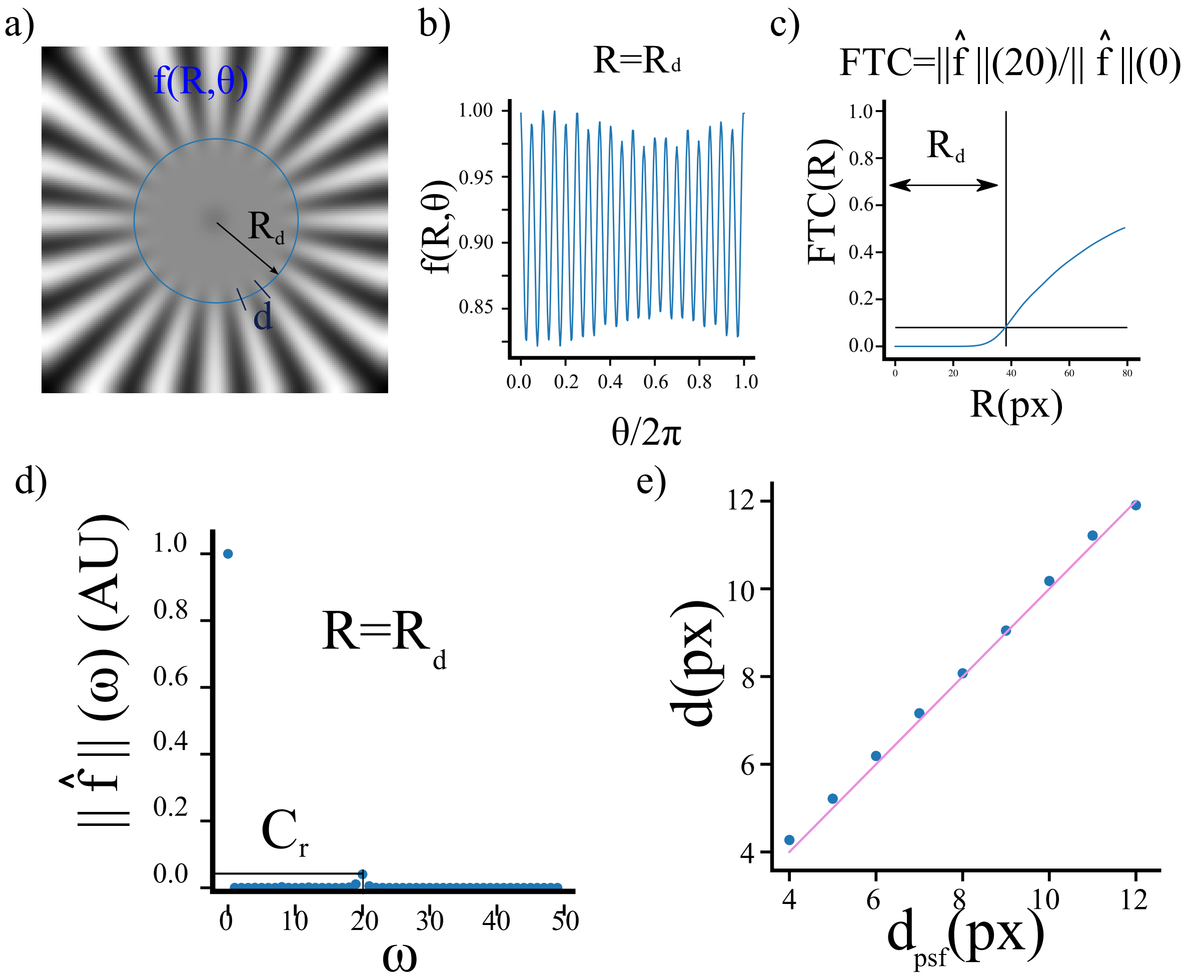

Supplementary Figure S 5. The resolution measurement process: a) To measure the resolution we convolve the siemens star with a convolution kernel of size d_{PSF} and we analyze the azimuthal profile $f(R,\theta)$ for different radious R. The radious $R_d = \pi/d_{PSF}10$ corresponds to the Rayleich criterion. b) The azimuthal profile $f(R,\theta)$ for $R = R_d$ c) is the Fourier transform contrast FTC as a function of the radious R. R_d is the radious in which $FTC = C_r$. d) is the Fourier spectrum of b. e) To justify the resolution measurement we convolved the siemens star with different convolution kernels of size d_{PSF} and measure the resolution d with the FTC criterion, here we show that the resolution measurement d is very close to the kernel size d_{PSF}.

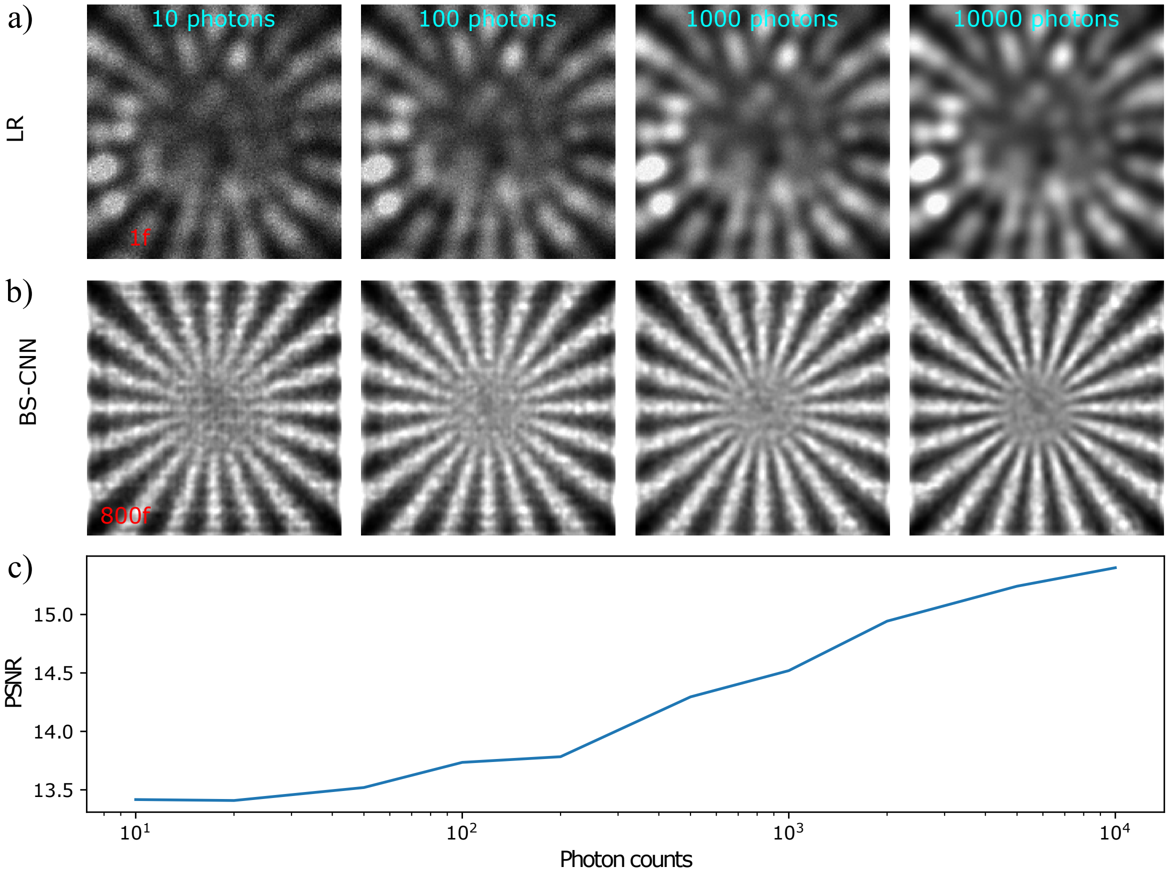

Supplementary Figure S 6. The performance of BS-CNN on noisy data: a) The Low resolution image LR is degraded with Poissonian and Gaussian noise. As the number of incident photons on the camera is increasing the signal to noise ratio is increasing. Here we show four different noise-degraded configurations for the same single low resolution image . b) The BS-CNN reconstruction corresponding to the different noise levels c) The peak signal to noise ration PSNR as a function of the photon counts

4 Resolution Measurement and Robustness to Noise

4.1 Resolution Measurement

In an optical system the resolution is limited due to diffraction[4]. The radious of the Airy disk defines the resolution. Two point objects that lie inside a disk with radius smaller than the Airy's disk and emit light at the same intensity can not be resolved. In fact, the intensity profile of a line that connects their center drops at about 25 per cent (Rayleigh criterion). When the resolution of an optical system is not known, one way to measure the resolution is to adjust two point light source up to the point that the line profile drops below the Rayleigh criterion. However, due to noise and pixelisation, and the difficulty in adjusting point-light sources, the resolution Rayleigh method may not always be the optimum. An alternative way, that we also discuss in the main text, is to use the approach of[5], as illustrated in Supplementary Fig. S5, where a periodic signal g is used as the ground truth object

$$g(\theta) = 1 + cos(20\theta) = ||\hat{g}_0|| + ||\hat{g}_{20}||cos(20\theta), \tag{7}$$

where θ is the angle from the center, the ˆ symbol is used for the Fourier transform and $||||$ is the absolute value. In the ground truth object the contrast

$$C_{gt} = 2\hat{g}(20)/\hat{g}(0) = (g_{max} - g_{min})/(g_{max} + g_{min}), \tag{8}$$

is always equal to unity. The low-resolution signal $f(R,\theta)$ is a convolution of $g(\theta)$ with an airy disk kernel of resolution d_{PSF}. The resolution in this setup is defined as

$$d = R_d 2\pi/20, \tag{9}$$

where R_d is the minimum radious in which the fourier transform contrast

$$FTC = 2||\hat{f}(20)||/||\hat{f}(0)||, \tag{10}$$

is above a constant C_r.

$$FTC \geq C_r. \tag{11}$$

We find numerically (Supplementary Fig S.5) that $C_r = 0.08$, by analyzing the azimuthal profiles of airy disk kernels of different resolution d_{PSF}. With this criterion, we define the resolution improvement in the main text to be the ratio d_{PSF}/d_{al}, where d_{al} is the value of Eq. 9 for different algorithms.

4.2 Robustness of BS-CNN to Noise

In order to investigate the effects of experimental imperfections and the robustness of our method, we perform numerical simulations that reproduce the sources of noise in the apparatus. In particular, they are the read out noise and the photon counting noise. The noisy low resolution images are prepared by treating the readout noise as a Gaussian distributed noise $\mathscr{N}(\mu, \sigma^2)$ with the mean μ corresponding at the camera offset and $\sigma = 1.7$ camera counts. The photon noise is a Poissonian noise $\mathscr{P}(N_{ph})$ which mean corresponds to the total number of photons N_{ph} arriving at the camera.

$$N_{ph} = 0.49\frac{(counts - 100)}{0.71} \tag{12}$$

Thus, the noisy LR image is

$$LR_{noisy} = \mathscr{P}(LR) + \mathscr{N}(100, 1.7) \tag{13}$$

We test our algorithm for different photon counts in Supplementary Fig. S 6. We find that BS-CNN reconstruct less homogeneous images but the with a good resolution improvement even to low light conditions.

References

1. Leonetti, M., Grimaldi, A., Ghirga, S., Ruocco, G. & Antonacci, G. Scattering Assisted Imaging. *Sci Rep* **9**, 4591, DOI: 10.1038/s41598-019-40997-6 (2019).
2. Mangeat, T. *et al.* Super-resolved live-cell imaging using random illumination microscopy. *Cell Reports Methods* **1**, 100009, DOI: https://doi.org/10.1016/j.crmeth.2021.100009 (2021).
3. Wang, Z., Bovik, A., Sheikh, H. & Simoncelli, E. Image Quality Assessment: From Error Visibility to Structural Similarity. *IEEE Transactions on Image Process.* **13**, 600–612, DOI: 10.1109/TIP.2003.819861 (2004).
4. Abbe, E. Beiträge zur Theorie des Mikroskops und der mikroskopischen Wahrnehmung. *Arch. für Mikroskopische Anat.* **9**, 413–468, DOI: 10.1007/BF02956173 (1873).
5. Mudry, E. *et al.* Structured illumination microscopy using unknown speckle patterns. *Nat. Photonics* **6**, 312–315, DOI: 10.1038/nphoton.2012.83 (2012).
